# Heat-related mortality in Europe during the summer of 2022

**DOI:** 10.1038/s41591-023-02419-z

**Published:** 2023-07-10

**Authors:** Joan Ballester, Marcos Quijal-Zamorano, Raúl Fernando Méndez Turrubiates, Ferran Pegenaute, François R. Herrmann, Jean Marie Robine, Xavier Basagaña, Cathryn Tonne, Josep M. Antó, Hicham Achebak

**Affiliations:** 1 ISGlobal, Barcelona, Spain; 2 Universitat Pompeu Fabra, Barcelona, Spain; 3 Medical School of the University of Geneva, Geneva, Switzerland; 4 Division of Geriatrics, Department of Rehabilitation and Geriatrics, Geneva University Hospitals, Thônex, Switzerland; 5 Molecular Mechanisms in Neurodegenerative Dementia, University of Montpellier, Montpellier, France; 6 École Pratique des Hautes Études, Institut National de la Santé et de la Recherche Médicale, Montpellier, France; 7 PSL Research University, Paris, France; 8 CIBER Epidemiología y Salud Pública, Barcelona, Spain; 9 Institut National de la Santé et de la Recherche Médicale, France Cohortes, Paris, France

**Keywords:** Epidemiology, Health policy

## Abstract

Over 70,000 excess deaths occurred in Europe during the summer of 2003. The resulting societal awareness led to the design and implementation of adaptation strategies to protect at-risk populations. We aimed to quantify heat-related mortality burden during the summer of 2022, the hottest season on record in Europe. We analyzed the Eurostat mortality database, which includes 45,184,044 counts of death from 823 contiguous regions in 35 European countries, representing the whole population of over 543 million people. We estimated 61,672 (95% confidence interval (CI) = 37,643–86,807) heat-related deaths in Europe between 30 May and 4 September 2022. Italy (18,010 deaths; 95% CI = 13,793–22,225), Spain (11,324; 95% CI = 7,908–14,880) and Germany (8,173; 95% CI = 5,374–11,018) had the highest summer heat-related mortality numbers, while Italy (295 deaths per million, 95% CI = 226–364), Greece (280, 95% CI = 201–355), Spain (237, 95% CI = 166–312) and Portugal (211, 95% CI = 162–255) had the highest heat-related mortality rates. Relative to population, we estimated 56% more heat-related deaths in women than men, with higher rates in men aged 0–64 (+41%) and 65–79 (+14%) years, and in women aged 80+ years (+27%). Our results call for a reevaluation and strengthening of existing heat surveillance platforms, prevention plans and long-term adaptation strategies.

## Abstract (web summary)

This ecological analysis using the Eurostat database estimated that summer 2022, the hottest summer on record, was associated with over 61,000 heat-related deaths across 35 countries in Europe, with the highest mortality rates in countries near the Mediterranean Sea.

## Main

Anthropogenic emissions of greenhouse gases have led to a detectable rise in global temperatures, which is associated with an increase in the frequency and intensity of heat waves and hot summers^1,2^. Globally, the last 8 years have been the warmest on record, and 2022 was the fifth warmest year^3^. In this context, Europe emerges as a major climatic hotspot^4,5^, given that warming since preindustrial levels is almost 1 °C higher than the corresponding global increase, and higher than in any other continent^6^. Moreover, climate change projections for the continent indicate that temperatures, and their health impacts, will rise at an accelerated rate unless strong mitigation and adaptation actions are put in place^7,8^.

Exposure to heat poses a major threat to high-risk populations in Europe and worldwide by substantially contributing to increased morbidity and mortality^9,10^. Heat waves are the extreme weather events with the highest impact in terms of attributable counts of death^11^. Heat-related mortality has been a major concern for the past two decades in Europe, especially after the 71,449 excess deaths registered during the months of June, July, August and September of 2003 (ref. ^12^). The resulting societal awareness of the short-term health effects of heat led to the design and implementation of heat prevention plans and other adaptation strategies to protect at-risk populations across the continent^13,14^, that is, older adults with preexisting cardiovascular and respiratory diseases^15–17^, women^18,19^ and socially isolated^20,21^ or socioeconomically disadvantaged^19,22^ individuals. Although there is some evidence that heat prevention plans, including preparedness and response strategies, intervention actions and heat-health early warning systems, can reduce the health burden of ambient temperatures, the evidence of their effectiveness is still limited^23^.

The summer of 2022 was the hottest season on record in Europe, characterized by an intense series of heat waves, which led to extremes in terms of temperature, drought and fire activity^24,25^. The record-breaking temperatures during the summer of 2022 were monitored by existing surveillance systems, activating an array of national and regional heat prevention and adaptation plans. The European Statistical Office, Eurostat, reported unusually high excess mortality rates for the summer of 2022 (ref. ^26^), but so far the heat-related mortality burden has not been quantified across the European continent. The aim of this study was to use epidemiological models to estimate the sex- and age-specific mortality burden associated with the record-breaking temperatures registered during the 14-week period between 30 May and 4 September 2022 (weeks 22–35). Moreover, we compared this mortality burden within the broader context of the summer of 2003 and the accelerated warming observed in the continent during the last decade (2013–2022).

## Results

### Association between temperature and mortality

**Fig. 1:**
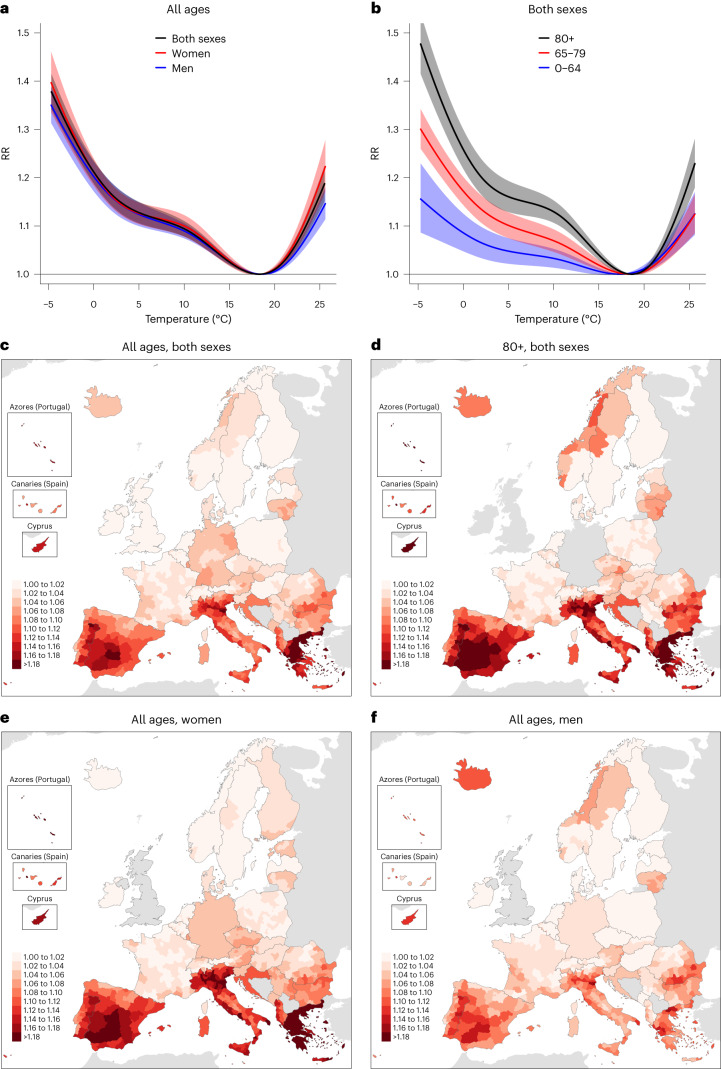
Temperature-related risk of death during 2015–2019. **a**,**b**, Cumulative relative risk of death (unitless) in Europe for the overall population (black), women (red) and men (blue) (**a**) and people aged 0–64 (blue), 65–79 (red) and 80+ (black) years (**b**), together with their 95% CIs (shadings). **c**–**f**, Regional relative risk of death (unitless) at the temperature 95th centile for the overall population (**c**), people aged 80+ years (**d**), women (**e**) and men (**f**).

**Extended Data Fig. 1:**
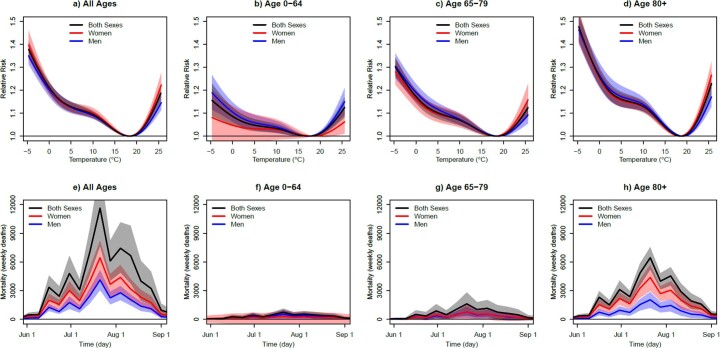
European sex- and age-specific temperature-related risk of death and heat-related mortality. Panels (a-d) depict the cumulative relative risk of death (unitless) in Europe during 2015-2019. Panels (e-h) display the weekly heat-related mortality (weekly deaths) aggregated over Europe during the summer of 2022. The shadings represent the 95% confidence intervals. All-age data by sex was not available in the United Kingdom (red and blue lines in panels a,e). Data by sex and age groups was not available in the United Kingdom, Ireland and Germany (panels b-d, f-h).

The cumulative temperature–mortality association in Europe shows a monotonically increasing risk of death for temperatures above and below the minimum mortality temperature (Fig. 1a,b; associations by sex and age groups available in Extended Data Fig. 1a–d). This optimum temperature was around 17–19 °C, with small differences according to sex (18.32 °C for women and 18.55 °C for men), but generally warmer values for older adults (17.39, 18.33 and 18.56 °C for the age groups 0–64, 65–79 and 80+ years, respectively). The slopes of the relative risk (RR) association for temperatures colder and hotter than the optimum temperature also increased with age (Fig. 1b); however, the age risk pattern differed according to sex (Extended Data Fig. 1b–d). On the one hand, the slope for colder temperatures was similar in women and men, except in the age group 0–64 years, in which the risk was higher in men. On the other hand, the slope for hotter temperatures was steeper in men aged 0–64 years and in women aged 65–79 and 80+ years. The overall spatial distribution of the RR at the temperature 95th centile emphasizes the latitudinal differences in heat-related mortality risk (Fig. 1c). Thus, the highest risks of heat-related mortality were observed in countries near the Mediterranean Sea in all sex and age groups, with generally higher values for older adults (Fig. 1d) and women (Fig. 1e,f).

### Temperatures and heat-related mortality numbers

**Fig. 2:**
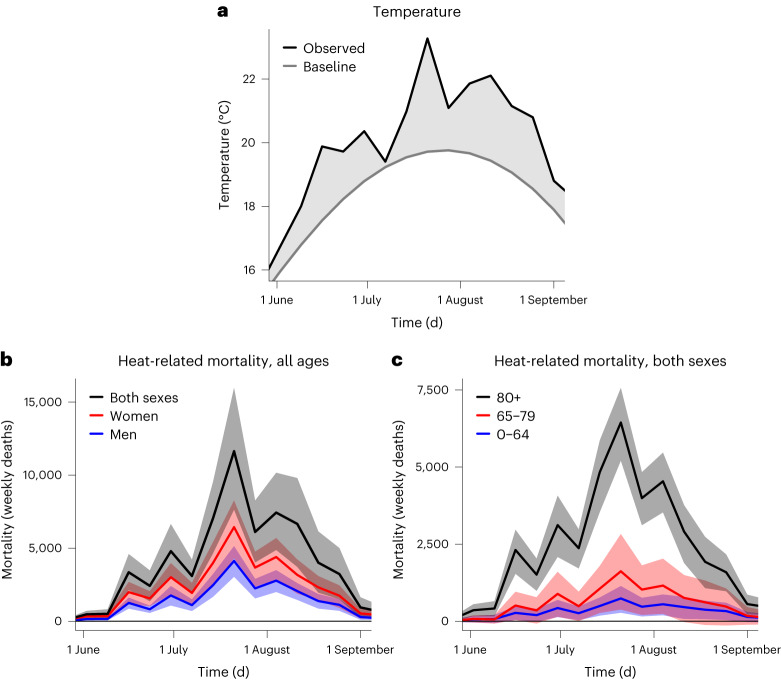
Weekly temperature and heat-related mortality numbers in Europe during the summer of 2022. **a**, Weekly baseline (gray line) and observed (black line) temperature (°C) averaged over Europe. Temperature anomalies are defined as the difference between observed and baseline temperatures (gray shading). Baseline temperatures were computed as the mean annual cycle of observed temperatures in the reference period 1991–2020. **b**,**c**, Weekly heat-related mortality (weekly deaths) aggregated over Europe for the overall population (black), women (red) and men (blue) (**b**) and people aged 0–64 (blue), 65–79 (red) and 80+ (black) years (**c**), together with their 95% CIs (shadings). The numbers for women and men in **b** do not include the United Kingdom; values for the age groups in **c** do not include Germany, Ireland and the United Kingdom.

**Extended Data Fig. 2:**
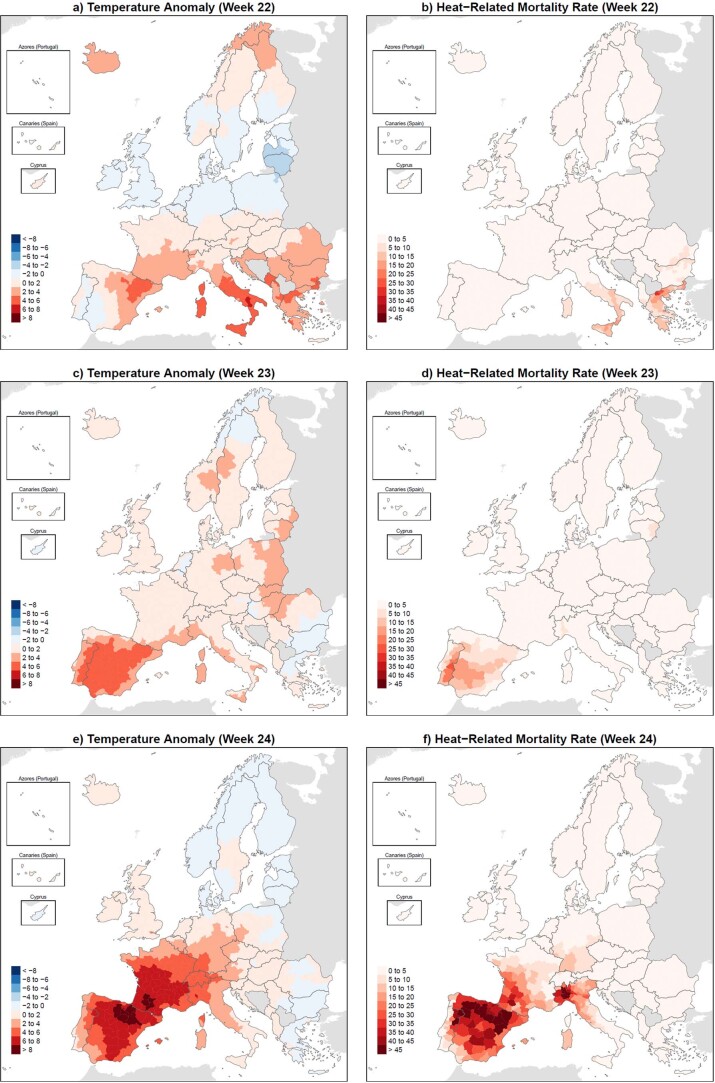
Regional temperature anomaly and heat-related mortality rate during weeks 22, 23 and 24. Regional temperature anomaly (a,c,e; °C) and heat-related mortality rate (b,d,f; weekly deaths per million) during weeks 22 (May 30 – June 5; a,b), 23 (June 6 – June 12; c,d) and 24 (June 13 – June 19; e,f) of the year 2022.

**Extended Data Fig. 3:**
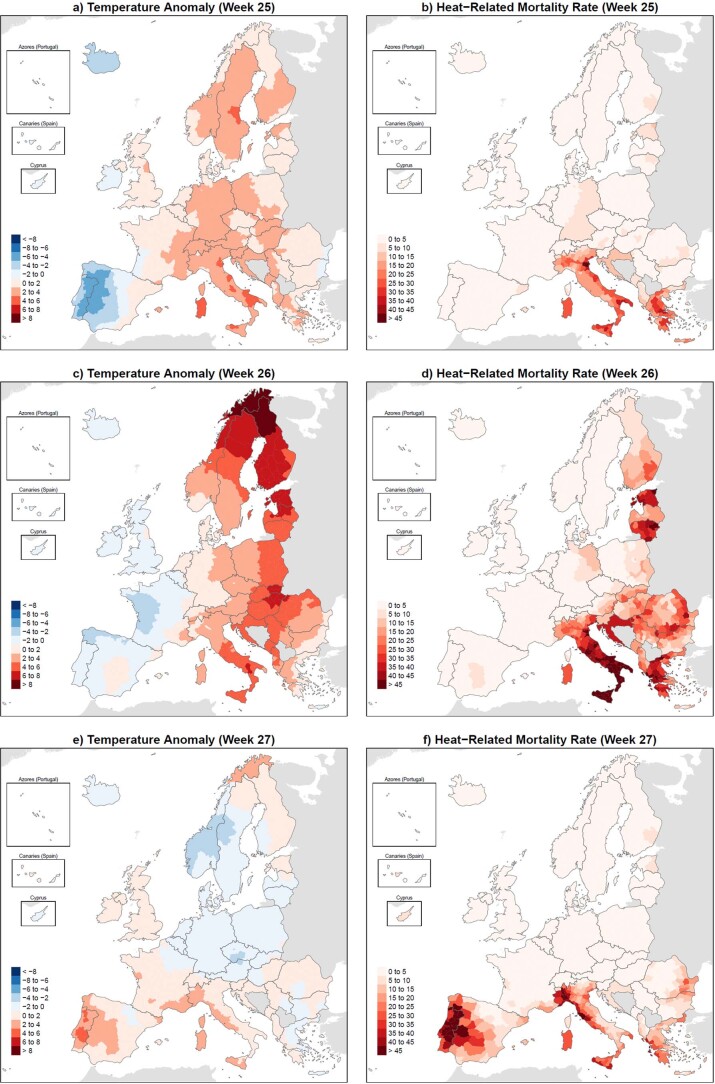
Regional temperature anomaly and heat-related mortality rate during weeks 25, 26 and 27. Regional temperature anomaly (a,c,e; °C) and heat-related mortality rate (b,d,f; weekly deaths per million) during weeks 25 (June 20 – June 26; a,b), 26 (June 27 – July 3; c,d) and 27 (July 4 – July 10; e,f) of the year 2022.

**Extended Data Fig. 4:**
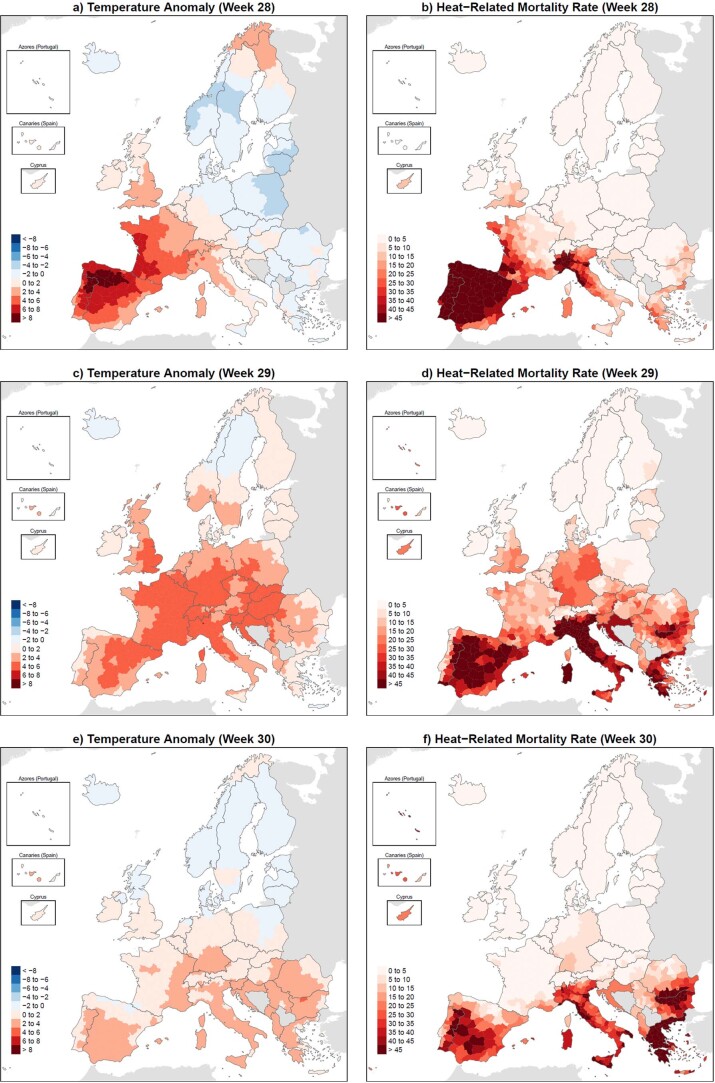
Regional temperature anomaly and heat-related mortality rate during weeks 28, 29 and 30. Regional temperature anomaly (a,c,e; °C) and heat-related mortality rate (b,d,f; weekly deaths per million) during weeks 28 (July 11 – July 17; a,b), 29 (July 18 – July 24; c,d) and 30 (July 25 – July 31; e,f) of the year 2022.

**Extended Data Fig. 5:**
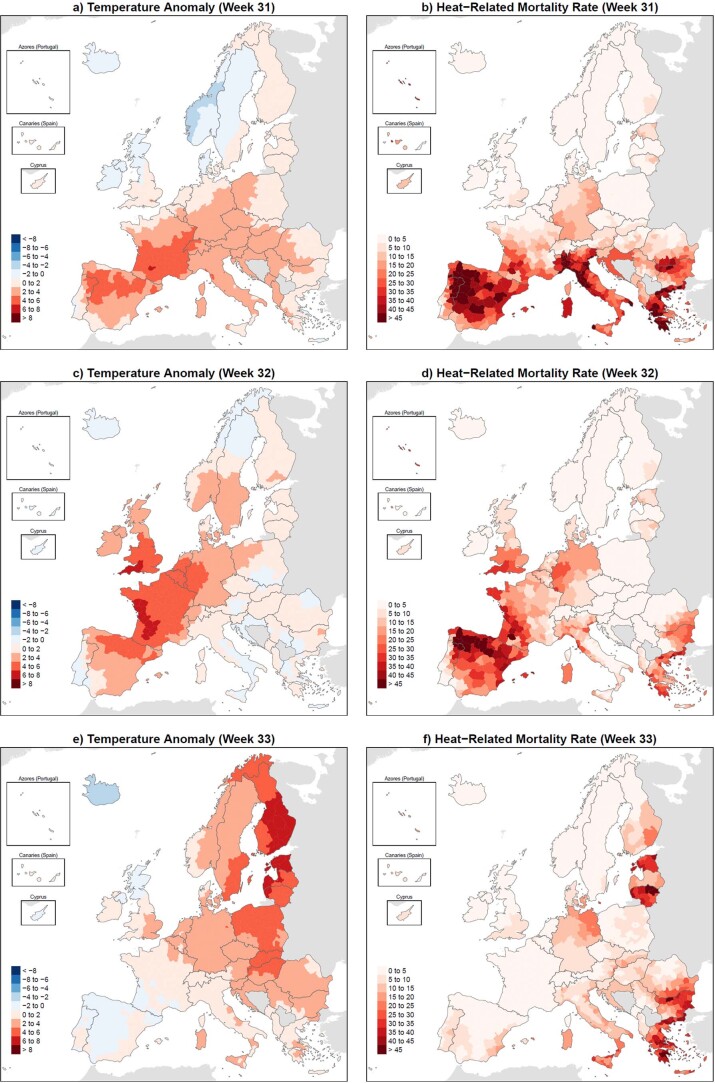
Regional temperature anomaly and heat-related mortality rate during weeks 31, 32 and 33. Regional temperature anomaly (a,c,e; °C) and heat-related mortality rate (b,d,f; weekly deaths per million) during weeks 31 (August 1 – August 7; a,b), 32 (August 8 – August 14; c,d) and 33 (August 15 – August 21; e,f) of the year 2022.

**Extended Data Fig. 6:**
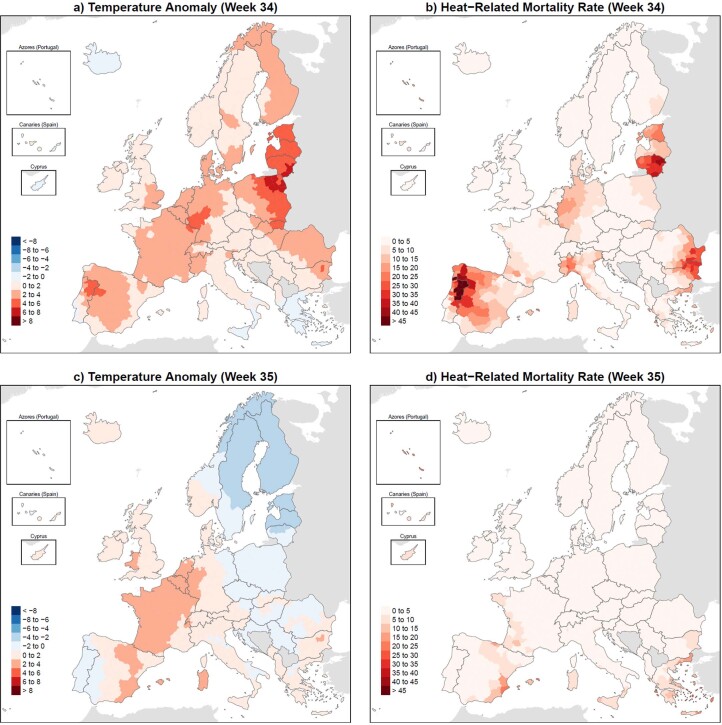
Regional temperature anomaly and heat-related mortality rate during weeks 34 and 35. Regional temperature anomaly (a,c; °C) and heat-related mortality rate (b,d; weekly deaths per million) during weeks 34 (August 22 – August 28; a,b) and 35 (August 29 – September 4; c,d) of the year 2022.

Observed European mean temperatures uninterruptedly exceeded the baseline climatological values of the 1991–2020 period in all of the weeks during summer 2022 (Fig. 2a). European weekly temperature anomalies in 2022 ranged between +0.78 and +2.33 °C in June (weeks 22–26), between +0.18 and +3.56 °C in July (weeks 26–30), and between +0.91 and +2.67 °C in August (weeks 31–35). Although several subcontinental warmer-than-average weeks were recorded during the summer (Extended Data Figs. 2–6), the most intense, pan-European heat wave was observed during week 29 (between 18 and 24 July); this week alone was estimated to be associated with 11,637 (95% confidence interval (CI) = 7,639–15,970) heat-related deaths (Fig. 2b), particularly in Central and Southern Europe (Extended Data Fig. 4c,d). The largest temperature anomalies coincided with the peak of the mean annual cycle of temperatures, that is, from mid-July to mid-August (Fig. 2a). The joint effect of the annual cycle and the anomalies magnified the mortality numbers and was associated with 38,881 (95% CI = 25,051–53,699) heat-related deaths between 11 July and 14 August (Fig. 2b). Heat-related mortality during this 5-week period accounted for nearly two-thirds of the overall summer (61,672; 95% CI = 37,643–86,807; Table 1) and annual (62,862; 95% CI = 37,935–88,780; Supplementary Table 1) heat-related mortality numbers. Italy (18,010; 95% CI = 13,793–22,225), Spain (11,324; 95% CI = 7,908–14,880), Germany (8,173; 95% CI = 5,374–11,018), France (4,807; 95% CI = 1,739–8,123), the United Kingdom (3,469; 95% CI = 370–6,676) and Greece (3,092; 95% CI = 2,217–3,915) were the countries with the highest summer heat-related deaths (Table 1).

**Table 1:** National sex-specific heat-related mortality numbers and rates during the summer of 2022

	Attributable number (deaths)			Attributable rate (deaths per million)		
Country	Overall	Women	Men	Overall	Women	Men
Albania	352 (97, 586)	186 (39, 336)	80 (4, 155)	117 (32, 195)	125 (26, 225)	53 (2, 102)
Austria	419 (109, 741)	274 (−55, 570)	199 (66, 332)	47 (12, 83)	60 (−12, 125)	45 (15, 75)
Belgium	434 (−26, 911)	264 (−68, 558)	159 (−26, 341)	38 (−2, 79)	45 (−12, 95)	28 (−5, 60)
Bulgaria	1,277 (549, 2,072)	678 (138, 1,145)	556 (239, 867)	176 (75, 285)	182 (37, 307)	157 (68, 245)
Switzerland	302 (48, 557)	255 (54, 433)	93 (20, 161)	35 (6, 64)	58 (12, 99)	22 (5, 37)
Cyprus	101 (24, 173)	56 (6, 110)	47 (17, 75)	113 (27, 193)	123 (13, 240)	107 (38, 171)
Czechia	279 (−25, 607)	290 (37, 520)	38 (−45, 122)	26 (−2, 56)	53 (7, 95)	7 (−8, 23)
Germany	8,173 (5,374, 11,018)	3,925 (1,656, 6,403)	2,771 (1,333, 4,149)	98 (64, 132)	93 (39, 152)	68 (32, 101)
Denmark	252 (42, 468)	119 (−51, 274)	59 (−19, 136)	43 (7, 80)	41 (−17, 93)	20 (−7, 47)
Estonia	167 (26, 296)	113 (7, 214)	39 (−5, 83)	123 (19, 217)	157 (9, 297)	61 (−8, 129)
Greece	3,092 (2,217, 3,915)	2,076 (1,551, 2,586)	822 (448, 1,186)	280 (201, 355)	367 (274, 457)	153 (83, 220)
Spain	11,324 (7,908, 14,880)	7,190 (4,426, 9,478)	4,250 (2,825, 5,633)	237 (166, 312)	295 (182, 389)	181 (121, 241)
Finland	225 (−94, 562)	278 (−15, 551)	30 (−14, 71)	40 (−17, 100)	98 (−5, 194)	11 (−5, 26)
France	4,807 (1,739, 8,123)	2,424 (−473, 4,964)	2,584 (1,237, 3,889)	73 (26, 124)	71 (−14, 146)	81 (39, 122)
Croatia	731 (346, 1,069)	469 (198, 708)	212 (72, 344)	172 (82, 252)	213 (90, 322)	104 (35, 168)
Hungary	513 (−126, 1,207)	529 (74, 915)	129 (−131, 396)	51 (−13, 121)	101 (14, 175)	27 (−27, 83)
Ireland	26 (−168, 199)	38 (−90, 174)	0 (0, 0)	5 (−34, 40)	15 (−35, 69)	0 (0, 0)
Iceland	0 (0, 0)	0 (−2, 3)	0 (0, 0)	0 (0, 0)	2 (−12, 17)	0 (0, 0)
Italy	18,010 (13,793, 22,225)	11,917 (8,078, 15,148)	6,268 (4,619, 7,817)	295 (226, 364)	379 (257, 482)	211 (156, 264)
Liechtenstein	1 (−2, 3)	1 (−1, 3)	0 (0, 0)	19 (−42, 73)	41 (−56, 143)	0 (−10, 11)
Lithuania	381 (158, 618)	157 (−13, 309)	190 (94, 282)	128 (53, 208)	99 (−8, 194)	138 (68, 204)
Luxembourg	44 (−1, 91)	25 (−1, 51)	7 (−7, 20)	69 (−2, 144)	79 (−3, 162)	22 (−21, 62)
Latvia	105 (−33, 242)	42 (−69, 144)	46 (−20, 111)	52 (−16, 120)	39 (−63, 133)	49 (−21, 120)
Montenegro	50 (−12, 108)	31 (−17, 83)	7 (−8, 21)	81 (−19, 173)	100 (−55, 262)	22 (−26, 69)
Malta	76 (−2, 150)	41 (−11, 90)	43 (12, 72)	147 (−5, 290)	166 (−43, 363)	160 (43, 270)
Netherlands	469 (−8, 981)	326 (−117, 727)	155 (−49, 357)	27 (0, 56)	37 (−13, 82)	18 (−6, 41)
Norway	30 (−32, 86)	8 (−43, 58)	28 (−2, 57)	5 (−6, 16)	3 (−16, 22)	10 (−1, 21)
Poland	763 (−283, 1860)	559 (−417, 1446)	259 (−73, 576)	20 (−7, 48)	28 (−21, 73)	14 (−4, 31)
Portugal	2,212 (1,703, 2,679)	1,227 (761, 1,618)	828 (592, 1,064)	211 (162, 255)	222 (138, 293)	166 (119, 214)
Romania	2,455 (1,201, 3,797)	1,130 (56, 2,145)	1,323 (779, 1,837)	122 (60, 189)	110 (5, 209)	135 (79, 187)
Serbia	574 (226, 955)	465 (244, 651)	253 (89, 415)	81 (32, 135)	129 (68, 180)	74 (26, 121)
Sweden	40 (−104, 200)	46 (−100, 181)	9 (−30, 50)	4 (−10, 19)	9 (−19, 35)	2 (−6, 10)
Slovenia	154 (−24, 307)	100 (−4, 209)	58 (−4, 119)	73 (−12, 146)	96 (−4, 200)	55 (−3, 112)
Slovakia	365 (62, 676)	164 (−5, 314)	128 (−12, 267)	66 (11, 123)	58 (−2, 111)	48 (−4, 99)
United Kingdom	3,469 (370, 6,676)	Not available	Not available	52 (6, 100)	Not available	Not available
**Europe**	**61,672 (37,643, 86,807)**	**35,406 (21,576, 46,634)**	**21,667 (14,684, 27,998)**	**114 (69, 160)**	**145 (89, 192)**	**93 (63, 120)**

Summer refers to the 14-week period between 30 May and 4 September 2022 (weeks 22–35). Values in parentheses represent the 95% CIs. The numbers and rates for women and men do not include the United Kingdom.

We estimated 63% more heat-related deaths in women (35,406; 95% CI = 21,576–46,634) than in men (21,667; 95% CI = 14,684–27,998) during the summer of 2022 (Table 1 and Supplementary Table 2). The death toll steeply increased with age, with 4,822 (95% CI = 1,130–8,158), 9,226 (95% CI = 665–17,382) and 36,848 (95% CI = 27,591–45,509) heat-related deaths in the age groups 0–64, 65–79 and 80+ years, respectively (Table 2). Italy, Spain, Germany and France had the highest female and male heat-related mortality numbers (Table 1). Italy had the highest number of heat-related deaths among the age groups 65–79 and 80+ years, but France had the highest number of heat-related deaths among people aged 0–64 years (Table 2). Despite differences in the magnitude of overall summer mortality numbers among different sex and age groups, the weekly changes in heat-related deaths were generally the same in all of them (Fig. 2b,c; weekly changes according to sex and age groups are available in Extended Data Fig. 1e–h). The differences according to sex largely varied with age, with higher heat-related deaths in men aged 0–64 years (+43%), but in women aged 65–79 (+6%) and 80+ (+121%) years (Supplementary Table 2).

**Table 2:** National age-specific heat-related mortality numbers and rates during the summer of 2022

	Attributable number (deaths)			Attributable rate (deaths per million)		
Country	0–64	65–79	80+	0–64	65–79	80+
Albania	24 (−18, 67)	48 (−19, 115)	165 (75, 246)	9 (−7, 26)	138 (−54, 330)	2,845 (1,283, 4,226)
Austria	52 (−27, 124)	160 (18, 303)	213 (−32, 472)	7 (−4, 17)	130 (15, 246)	423 (−63, 939)
Belgium	67 (−54, 171)	56 (−229, 324)	357 (113, 599)	7 (−6, 18)	34 (−141, 199)	554 (176, 931)
Bulgaria	108 (10, 197)	445 (135, 746)	737 (255, 1,237)	20 (2, 37)	384 (116, 644)	2,271 (787, 3,812)
Switzerland	34 (−68, 125)	67 (−126, 255)	230 (−71, 542)	5 (−10, 18)	56 (−106, 214)	487 (−151, 1,149)
Cyprus	9 (−6, 23)	15 (−8, 37)	72 (16, 119)	12 (−8, 31)	135 (−67, 325)	2,018 (459, 3,334)
Czechia	14 (−25, 49)	41 (−61, 144)	236 (12, 465)	2 (−3, 6)	24 (−35, 84)	522 (27, 1,030)
Germany	Not available	Not available	Not available	Not available	Not available	Not available
Denmark	43 (2, 83)	70 (−74, 215)	77 (−64, 218)	9 (0, 18)	78 (−82, 237)	265 (−219, 747)
Estonia	28 (16, 37)	32 (−10, 73)	71 (3, 138)	26 (15, 35)	167 (−52, 379)	885 (33, 1,719)
Greece	158 (6, 305)	321 (−10, 649)	2,245 (1437, 3054)	20 (1, 38)	198 (−6, 401)	2,977 (1,905, 4,050)
Spain	796 (189, 1,357)	1,476 (357, 2,544)	9,436 (5,855, 12,563)	21 (5, 36)	222 (54, 383)	3,273 (2,031, 4,357)
Finland	18 (−14, 49)	67 (−22, 156)	107 (−124, 337)	4 (−3, 11)	71 (−24, 164)	325 (−375, 1,019)
France	1,007 (171, 1,747)	1,673 (−87, 3,443)	2,832 (4, 5,395)	19 (3, 34)	169 (−9, 348)	706 (1, 1,346)
Croatia	30 (−43, 94)	160 (26, 289)	467 (189, 712)	10 (−14, 31)	244 (40, 440)	2,209 (892, 3,364)
Hungary	17 (−36, 69)	255 (−48, 547)	449 (15, 891)	2 (−5, 9)	165 (−31, 354)	1,009 (35, 2,000)
Ireland	Not available	Not available	Not available	Not available	Not available	Not available
Iceland	2 (−14, 17)	1 (−11, 11)	0 (0, 0)	6 (−44, 53)	14 (−264, 255)	0 (0, 0)
Italy	965 (236, 1,670)	2,326 (1,026, 3,601)	14,821 (12,004, 17,483)	21 (5, 37)	244 (108, 377)	3,290 (2,664, 3,880)
Liechtenstein	0 (0, 0)	0 (−1, 1)	0 (−1, 1)	2 (−8, 11)	38 (−111, 186)	147 (−577, 792)
Lithuania	77 (27, 123)	80 (4, 154)	211 (61, 367)	34 (12, 55)	199 (9, 383)	1,334 (383, 2,325)
Luxembourg	7 (−1, 15)	6 (−7, 19)	17 (−12, 42)	13 (−2, 28)	87 (−99, 265)	657 (−467, 1,636)
Latvia	11 (−13, 30)	9 (−36, 53)	129 (30, 221)	7 (−9, 20)	34 (−128, 190)	1,135 (261, 1,949)
Montenegro	5 (−9, 17)	12 (−9, 33)	25 (−10, 55)	9 (−17, 32)	146 (−120, 412)	1,248 (−496, 2,752)
Malta	14 (0, 30)	15 (−12, 39)	43 (−11, 84)	34 (−1, 70)	189 (−151, 498)	1,895 (−473, 3,740)
Netherlands	143 (1, 286)	129 (−303, 582)	306 (161, 440)	10 (0, 20)	48 (−113, 218)	359 (188, 516)
Norway	84 (−153, 270)	43 (−62, 146)	0 (−7, 8)	19 (−34, 61)	57 (−83, 196)	1 (−28, 33)
Poland	275 (−362, 844)	70 (−188, 328)	525 (205, 829)	9 (−12, 28)	13 (−35, 61)	310 (121, 489)
Portugal	192 (76, 303)	379 (129, 617)	1,464 (937, 1,947)	24 (10, 38)	219 (74, 357)	2,036 (1,302, 2,706)
Romania	457 (131, 757)	780 (67, 1,466)	1,186 (318, 2,108)	30 (9, 49)	273 (23, 513)	1,400 (375, 2,489)
Serbia	112 (15, 206)	300 (94, 506)	174 (−36, 394)	21 (3, 38)	264 (83, 445)	560 (−115, 1,269)
Sweden	22 (−27, 64)	64 (−118, 252)	16 (−76, 114)	3 (−3, 8)	41 (−76, 161)	29 (−136, 204)
Slovenia	9 (−26, 39)	34 (−18, 85)	94 (−11, 186)	5 (−15, 23)	105 (−54, 260)	798 (−91, 1,580)
Slovakia	42 (−3, 83)	90 (−99, 275)	145 (−36, 337)	9 (−1, 19)	119 (−130, 362)	790 (−195, 1,834)
United Kingdom	Not available	Not available	Not available	Not available	Not available	Not available
**Europe**	**4,822 (1,130, 8,158)**	**9,226 (665, 17,382)**	**36,848 (27,591, 45,509)**	**16 (4, 27)**	**160 (12, 302)**	**1,684 (1,261, 2,080)**

Summer refers to the 14-week period between 30 May and 4 September 2022 (weeks 22–35). The values in parentheses represent the 95% CIs. The numbers and rates do not include Germany, Ireland and the United Kingdom.

### Temperatures and heat-related mortality rates

**Fig. 3:**
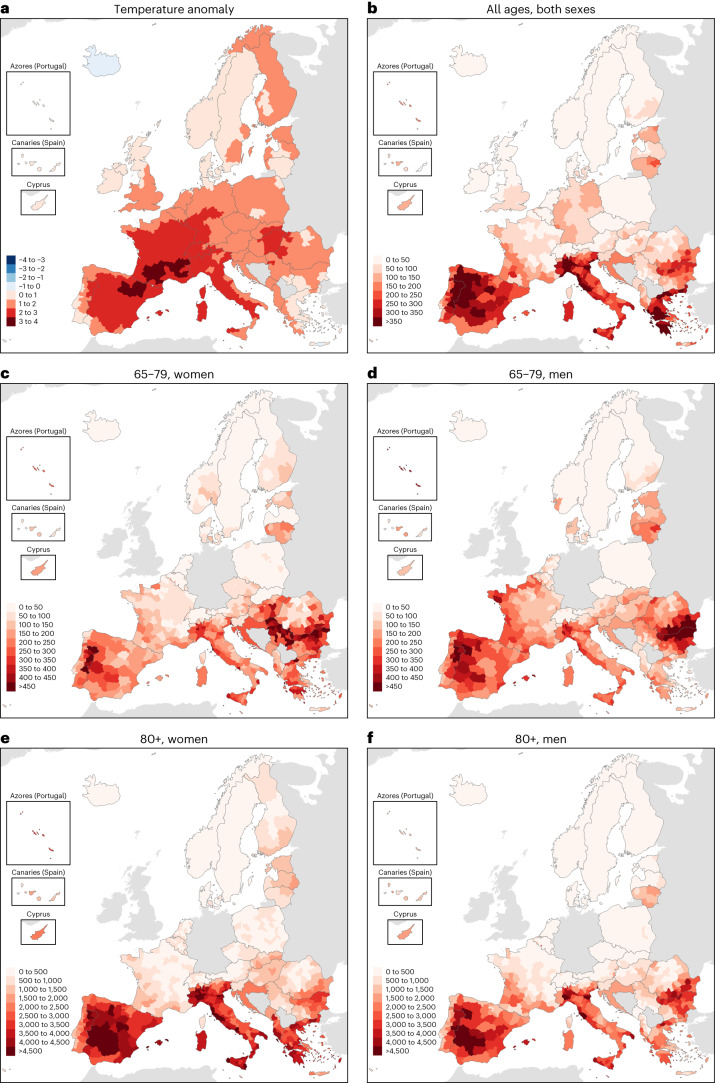
Regional temperature anomaly and heat-related mortality rate during the summer of 2022. **a**, Regional temperature anomaly (°C) averaged over the summer. **b**–**f**, Regional heat-related mortality rate (summer deaths per million) aggregated over the summer for the whole population (**b**), women aged 65–79 years (**c**), men aged 65–79 years (**d**), women aged 80+ years (**e**) and men aged 80+ years (**f**). Summer refers to the 14-week period between 30 May and 4 September 2022 (weeks 22–35).

Recorded summer mean temperatures exceeded the baseline climatological values in all European countries with the only exception being Iceland (−0.43 °C; Fig. 3a). The warmest summer temperature anomalies were mainly registered in Southwestern Europe, with the highest national values in France (+2.43 °C), Switzerland (+2.30 °C), Italy (+2.28 °C), Hungary (+2.13 °C) and Spain (+2.11 °C). The highest summer heat-related mortality rates were found in countries near the Mediterranean Sea, that is, Italy (295 deaths per million, 95% CI = 226–364), Greece (280, 95% CI = 201–355), Spain (237, 95% CI = 166–312) and Portugal (211, 95% CI = 162–255; Table 1), as well as in specific regions in Bulgaria, Romania, Croatia and Southern France (Fig. 3b). Overall, we estimated 114 (95% CI = 69–160) heat-related deaths per million in Europe during the summer, with 145 (95% CI = 89–192) female and 93 (95% CI = 63–120) male deaths per million (Table 1). The heat-related mortality rate also steeply increased with age, with 16 (95% CI = 4–27), 160 (95% CI = 12–302) and 1,684 (95% CI = 1,261–2,080) deaths per million in the age groups 0–64, 65–79 and 80+ years, respectively (Table 2). The differences according to sex largely varied with age, with higher heat-related mortality rates in men aged 0–64 (+41%) and 65–79 (+14%) years, and in women aged 80+ years (+27%) and for all age groups combined (+56%; Supplementary Table 2). These age-dependent sex differences were found in most countries with, for example, higher heat-related mortality rates in men than in women for the age group 65–79 years (Fig. 3c,d), but higher in women than in men for people aged 80+ years (Fig. 3e,f).

### Climate change and the summer of 2022

**Fig. 4:**
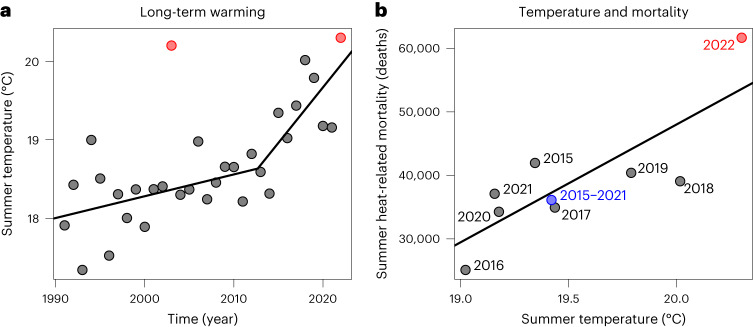
The summer of 2022 within the context of rising temperatures in Europe. **a**, Year-to-year time series of summer mean temperatures (°C) averaged over the analyzed European countries. The straight lines depict the linear fitting for the 1991–2012 (excluding the year 2003) and 2013–2022 periods. **b**, Relationship between summer mean temperature (°C) and summer heat-related mortality (summer deaths) in the analyzed European countries. The straight line shows the linear fitting for the 2015–2022 period.

On average over the 35 European countries analyzed here, the summer of 2022 was the warmest season on record (20.30 °C = *µ* + 2.51 *σ*), which exceeded both the summer of 2003 (20.20 °C = *µ* + 2.36 *σ*) and the threshold of 2.5 s.d. (*σ* = 0.67 °C) over the mean (*µ* = 18.62 °C) of the distribution of summer mean temperatures during the 1991–2020 period. The summer of 2003 was an exceptionally warm season within a period of relatively constant global and continental temperatures, commonly referred to as the global warming hiatus of 1998–2012 (ref. ^27^) (Fig. 4a). During the last decade (2013–2022), however, summer mean temperatures in the analyzed European countries sped up at an approximately constant rate of +0.142 °C per year, compared with the modest rate of +0.028 °C per year in 1991–2012 (the slopes in Fig. 4a). In that regard, although summer mean temperatures in 2022 followed the trend observed in the last decade (Fig. 4a), this was associated with an increase in 25,561 summer heat-related deaths compared to 2015–2021 (Fig. 4b). In this regard, we estimated that the warming observed since 2015 was associated with 18,547 additional summer heat-related deaths for every +1 °C increase in temperature (the slope in Fig. 4b), or in relative terms, 35.3 additional summer heat-related deaths per million for every +1 °C increase in temperature. Moreover, in the absence of adaptation to future summer warming, and by forward extrapolating the linear fittings in Fig. 4a,b, we would expect a heat-related mortality burden of 68,116 deaths on average every summer by the year 2030, 94,363 deaths by 2040 and 120,610 deaths by 2050.

### Sensitivity analyses

**Extended Data Fig. 7:**
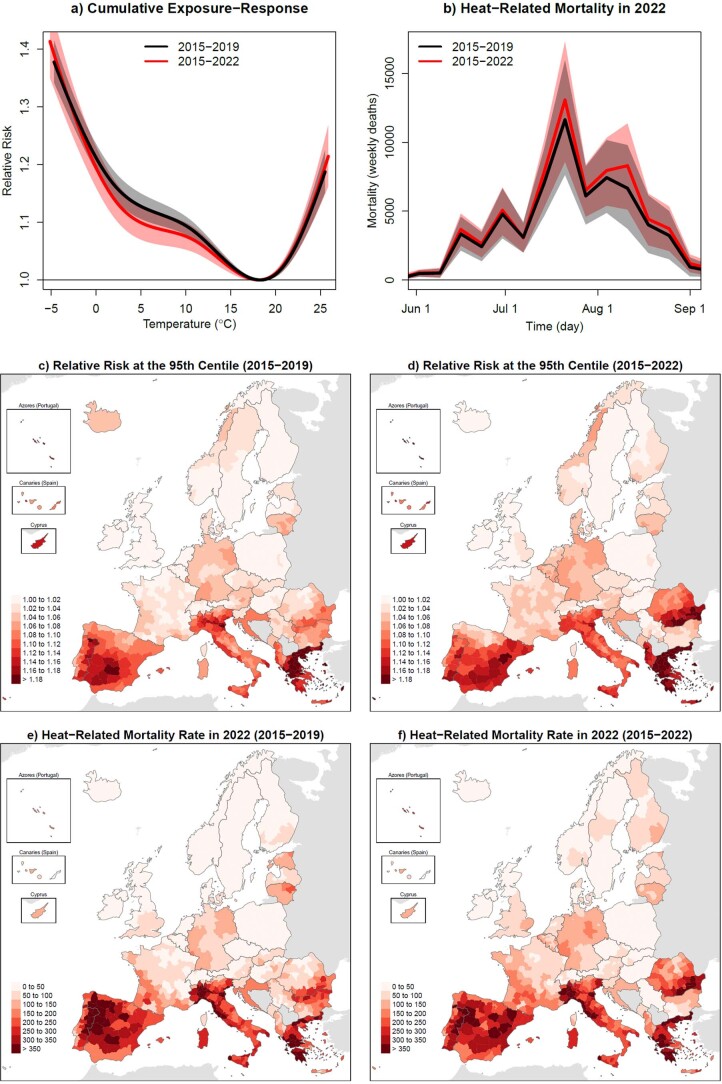
Sensitivity to the period of calibration of the epidemiological associations. Panels (a,b) depict the cumulative relative risk of death (unitless) in Europe (a) and the weekly heat-related mortality (weekly deaths) aggregated over Europe during the summer of 2022 (b), together with their 95% confidence intervals (shadings). Panels (c-f) display the regional relative risk of death (unitless) at temperature 95^th^ percentile (c,d) and the regional heat-related mortality rate (summer deaths per million) aggregated over the summer of 2022 (e,f). Values were obtained from epidemiological models calibrated with data from the periods 2015-2019 (black in a,b; and maps in c,e) and 2015-2022 (red in a,b; and maps in d,f). Summer refers to the 14-week period between May 30^th^ and September 4^th^ of 2022 (weeks 22-35).

We performed sensitivity analyses by modifying the modeling choices (Methods). In the main results, we calibrated the epidemiological models with temperature and mortality data from January 2015 to December 2019 to avoid any eventual interfering effect of the coronavirus disease 2019 pandemic. However, sensitivity analyses showed that estimates of the RRs and heat-related mortality during the summer (68,476 deaths; 95% CI = 44,884–90,803) and year (70,997; 95% CI = 46,612–94,777) of 2022 were only slightly higher when the pandemic period was included in the calibration of the epidemiological models, that is, 2015–2022 (Supplementary Table 1 and Extended Data Fig. 7).

**Extended Data Fig. 8:**
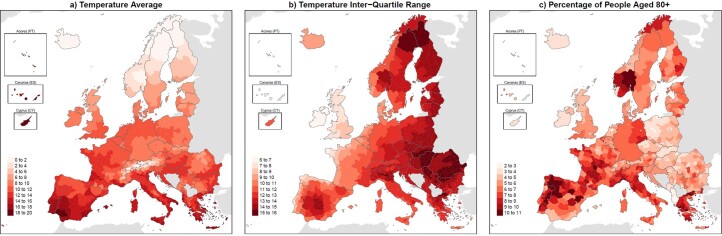
Meta-predictors included in the meta-regression. Maps correspond to the regional temperature average (a, °C), temperature inter-quartile range (b, °C) and percentage of people aged 80+ years (c, %).

The statistical analysis was done in two steps (Methods). In the first stage, we used quasi-Poisson regression models to calculate the location-specific temperature–lag–mortality relationship in each European region. We tested different configurations of the exposure–response function, being estimates of the heat-related mortality not sensitive to these choices (Supplementary Table 1). In the main results, we used the model configuration that better fitted the data based on the Akaike information criterion. In the second stage, we used a multivariate, multilevel meta-regression analysis to pool the location-specific coefficients obtained in the first step, including (1) country random effects and (2) the location-specific temperature average, (3) the temperature interquartile range and (4) the percentage of people aged 80+ years as meta-predictors (Extended Data Fig. 8). We tested these meta-predictors and we found that they explained a significant fraction of the spatial heterogeneity (Supplementary Table 3).

## Discussion

This European-wide study quantified the mortality associated with record-breaking temperatures during the summer of 2022, currently the hottest season on record in Europe, and analyzed it within the broader context of the summer of 2003 and the accelerated warming observed in the continent during the last decade (2013–2022). Epidemiological models were applied to a mortality database representing the whole population of over 543 million people from 823 contiguous regions in 35 European countries. Overall, we estimated 62,862 heat-related deaths in Europe in 2022; 61,672 of those deaths occurred between 30 May and 4 September. Italy, Spain, Germany, France, the United Kingdom and Greece had the highest summer heat-related mortality numbers. In relative terms, the largest summer heat-related mortality rates were found in countries near the Mediterranean Sea, which included Italy, Greece, Spain and Portugal. The results showed that there was a large increase in heat-related mortality during June–August 2022, approaching the record-breaking excess mortality of June–September 2003.

However, the comparison with 2003 needs to be interpreted with caution, given the large methodological differences in our study compared to previous estimates^12^. First, the estimate of the death toll during the summer of 2003 from Robine et al.^12^ was based on excess mortality numbers, which quantify the deviation of the mortality from the seasonally varying expected mortality from a reference period. As heat-related mortality occurs every summer, the estimate of excess deaths may exclude part of the heat-related mortality burden. On the other hand, this study used epidemiological models to quantify deaths specifically attributable to heat; therefore, our estimate is not expected to include mortality cases in which heat was not a contributor. Furthermore, we used weekly temperature and mortality data in our epidemiological models, which is expected to underestimate the day-to-day variability of the time series and possibly their lagged short-term associations. This is especially the case for heat, given that the risk of death is acute and generally does not last for more than 5 days^7,28,29^. As a reference, a previous study^30^ applied similar epidemiological models to daily temperature and mortality data for Spain only, and found that the summer heat-related mortality burden was 6% higher (that is, 12,054 deaths) than the one reported here for the same country (that is, 11,324 deaths). Finally, the excess mortality estimates during the summer of 2003 from Robine et al.^12^ were based on data from only 16 European countries, representing a population of nearly 400 million people^31,32^. As a reference, when restricted to the same regions and countries, our heat-related mortality estimates for the summer of 2022 (that is, 61,672 deaths) were 15% lower (that is, 52,121 deaths).

Furthermore, the warming levels and trends observed immediately before the summers of 2003 and 2022 were also very different. The summer of 2003 was an exceptionally rare event, even if the observed long-term anthropogenic warming was taken into account^33^. The exceptional nature of the event highlighted the shortcomings (or the inexistence) of heat prevention plans during the time, the fragility of health systems in dealing with climate-related health emergencies and the lack of awareness of the associated risks and impacts by the media and general population^13,21^. Instead, the temperatures in the summer of 2022 were not exceptional, in the sense that this could have been anticipated by forward extrapolation of the accelerated warming pathway observed during the last decade (2013–2022). Yet, this was associated with an increase of over 25,500 summer heat-related deaths compared to the 2015–2021 period. The rate of warming observed during the last decade emphasizes the urgent need for reevaluation and strengthening of adaptation strategies. Indeed, in the absence of further adaptation to the summer heat, and by forward extrapolating the linear fittings in Fig. 4a,b, we would expect a rapid increase to unprecedented summer heat-related mortality numbers in the coming years. However, the exceptional nature of 2003, with summer mean temperatures more than +2 °C warmer than the values expected from previous summers (Fig. 4a), leads to the speculation of what would have been the mortality burden during the summer of 2022 if a similar temperature anomaly had occurred. Although future studies are needed to answer this question, the possibility that a severe thermal anomaly with regard to the current warmer climate could produce an impact on mortality greater than the one observed in 2022 needs to be carefully considered in the next summers.

Although previous studies showed that the risk of death due to heat has decreased in several European countries^13,18,34^, our results suggest that past efforts toward an effective early adaptation response to observed warming, including preparedness and response strategies, intervention actions and heat-health early warning systems, had largely been insufficient to prevent the large magnitude of the heat-related mortality estimated for the summer of 2022. Despite the experience accumulated since 2003 (ref. ^23^), and the excess mortality estimates captured by EuroMOMO and Eurostat in several European countries, the magnitude of the overall death toll received relatively little attention. Despite the fact that many European countries activated heat prevention plans during the summer of 2022, the estimation of over 60,000 heat-related deaths suggests that prevention plans were only partially effective.

The burden of heat-related mortality was higher among women. Relative to population, we estimated 56% more heat-related deaths in women than in men, with higher rates in men aged 0–64 and 65–79 years, and in women aged 80+ years. Physiological differences and sociocultural factors have been suggested as potential explanations for these gaps^35–37^, but we also found that differences in age structure between men and women partly explained the higher risk for women at advanced ages and for men at younger ages. Prevention plans should also target a reduction of sex, age and other drivers of inequalities in the risk of heat-related mortality.

This study included an analysis of heat-related risks and mortality numbers according to sex and age groups, showing generally higher values in women and, as expected, steeply increased with age. We showed that age composition was a driver of between-sex and between-country differences^38–40^; this could closely relate to policy questions around how to efficiently protect the population from summer heat. Exposure to extreme heat, especially during summers such as in 2022, may differentially exacerbate preexisting or chronic risks among women and men in each age group. This raises questions about whether the observed sex differences in heat-related mortality risks and numbers are driven by differences or disparities, and what this would mean for policy, which is beyond the scope of the study and its methodological design. The combination of sex and age, socioeconomic level, education and underlying health status could have contributed to the magnitude and distribution of heat-related mortality in our study^41,42^. Unfortunately, with the current data availability in Europe, the analysis of the societal determinants of vulnerability and adaptation will require a larger effort^43^. Improving data availability for more granular pan-European studies to monitor the health and inequity dimensions of climate change should also be considered as part of the societal response to the climate crisis, as already emphasized by Robine et al. 15 years ago^12^.

Temperatures during the summer of 2022 were warmer than average in most of Europe, but the largest summer heat-related mortality rates were found in countries near the Mediterranean Sea. We showed that this latitudinal pattern closely resembled the spatial distribution of RRs, rather than the distribution of temperature anomalies (Figs. 1c and 3a,b). This mismatch was already observed during the summer of 2003, with the warmest summer mean temperature anomalies in Central Europe, mainly in France and Switzerland^44^, but the largest excess mortality in Spain (+13.7%), France (+11.8%) and Italy (+11.6%)^12^. Our study therefore emphasized the vulnerability of populations in Southern Europe^45^. As a major climate change hotspot^4,5^, these populations will be increasingly exposed to extreme summer conditions^2^ and would therefore be expected to experience increasingly higher heat-related mortality in the future^7,8^. Addressing geographical inequalities in current and future vulnerability to heat will also need to be prioritized by national and European governments and agencies.

This study has some limitations worth acknowledging. First, we used weekly temperature and mortality data in the epidemiological models, which is expected to underestimate the heat-related mortality of the summer of 2022. The scientific question of characterizing the biases of epidemiological models applied to weekly temperature and mortality data was beyond the scope of the present work and it will be addressed in a follow-up study. Second, data according to sex and age groups were not available in a reduced number of countries, that is, the United Kingdom, Ireland and Germany, which emphasized the urgent need to coordinate national agencies for statistics to create protocols integrating and homogenizing health data sources and improving open-access data for research, translation and policymaking. In our opinion, this should be a priority in the agenda of European governments, both from inside and outside the European Union, given that health data are managed at the country level and only integrated as open-access data in exceptional cases. Finally, the study analyzed records of all-cause mortality because cause-specific data were not available. This limitation is important because some of the differences in heat-related mortality risks and numbers between sex and age groups could be better interpreted within the framework of an in-depth analysis of the causes of death.

Due to global warming, temperatures in Europe are rising at a faster rate than the global average^6^. Taking into account the magnitude of heat-related mortality on the continent, our results call for a reevaluation and strengthening of heat surveillance platforms, prevention plans and long-term adaptation strategies. The high heat-related mortality that Europe experienced during the summer of 2022 calls for national governments and relevant agencies in the European Union and continental levels to increase the ambition and effectiveness of heat prevention and adaptation plans with urgency.

## Methods

### Data sources

We obtained weekly counts of all-cause mortality according to sex and age groups from Eurostat^46^. Missing data were complemented by contacting the corresponding national agencies for statistics. The final dataset included 45,184,044 counts of death (22,000,519 for women and 21,913,050 for men) between January 2015 and November 2022 from 823 contiguous regions representing over 543 million Europeans in 35 countries, namely Albania (12 regions), Austria (35), Belgium (44), Bulgaria (28), Croatia (1), Cyprus (1), Czechia (14), Denmark (11), Estonia (5), Finland (19), France (96), Germany (16), Greece (52), Hungary (20), Iceland (2), Ireland (1), Italy (103), Latvia (6), Liechtenstein (1), Lithuania (10), Luxembourg (1), Malta (1), Montenegro (1), the Netherlands (40), Norway (11), Poland (73), Portugal (25), Romania (42), Serbia (25), Slovakia (8), Slovenia (1), Spain (59), Sweden (21), Switzerland (26), and the United Kingdom (12). On average, each region represented a population of 660,000 Europeans. All-age data according to sex was not available in the United Kingdom and only at the country level in Germany. Data according to sex and age groups was not available for Germany, Ireland and the United Kingdom.

We transformed the hourly gridded 2-m temperature data from the high-resolution ERA5-Land reanalysis^47^ into weekly regional averages of daily mean 2-m temperature.

### Statistical analysis

In a nutshell, we used the regional temperature and mortality time series for the period from January 2015 to December 2019 to calibrate the epidemiological models, which were then used to transform the temperature and mortality time series from January 2015 to November 2022 into the weekly and summer heat-related mortality numbers over the years 2015–2022. Epidemiological models were fitted separately for each combination of sex and age groups.

More specifically, the statistical analysis was done in two stages. In the first stage, we used quasi-Poisson regression models, which allow for overdispersed counts of deaths, to calculate the location-specific temperature–lag–mortality relationship in each European region^28,29,48^. The models included (1) an intercept, (2) a natural cubic spline of time with 8 d.f. per year to control for the seasonal and long-term trends, and (3) a cross-basis function to estimate the exposure–lag–response association between weekly temperatures (temp) and mortality counts (mort):

$$\begin{array}{l}{\mathrm{log}}({E}({\mathrm{mort}}))=\\{\mathrm{intercept}}+{\mathrm{ns}}({\mathrm{time}},8 \;{\mathrm{d.f}}.\,{\mathrm{per}}\,{\mathrm{year}})+{\mathrm{crossbasis}}({\mathrm{temp}};0,1,2,3\,{\mathrm{weeks}})\end{array}$$

The lag–response function of the cross-basis was modeled with integer lag values of 0, 1, 2 and 3 weeks, and the exposure–response function with a natural cubic spline with three internal knots at the 10th, 50th and 90th centiles of the location-specific weekly temperature distribution.

In the second stage, we used a multivariate, multilevel meta-regression analysis^49^ to pool the location-specific coefficients obtained in the first stage. The meta-regression included (1) the country random effects, (2) the location-specific temperature average, (3) the temperature interquartile range and (4) the percentage of people aged 80+ years as meta-predictors (Extended Data Fig. 8)^50^. We derived the best linear unbiased predictions of the temperature–mortality relationship in each region from the meta-regression^51^ to obtain the location-specific minimum mortality temperature and to transform the regional temperature and mortality time series from January 2015 to November 2022 into the weekly and summer heat-related mortality numbers over the years 2015–2022 (ref. ^52^). Heat-related mortality was calculated for the weeks with average temperatures above the location-specific minimum mortality temperature^18^. Regional heat-related mortality numbers were aggregated to obtain the national and European burdens^7,8^. Similarly, we computed 1,000 Monte Carlo simulations of the regional heat-related mortality numbers and separately aggregated the numbers in each simulation to calculate the 95% CIs at the national and continental levels^28,29,50^. We calculated heat-related mortality rates (in deaths per million) by using the yearly regional population estimates from Eurostat^53^.

We defined the temperature anomalies as the difference between observed and baseline temperatures. The baseline temperature was computed as the mean annual cycle of observed temperatures in the reference period 1991–2020, calculated as a linear regression model with temperature as the dependent variable, and (1) an intercept and (2) a natural cubic spline of day of the year with 6 d.f. as independent variables.

### Reporting summary

Further information on research design is available in the Nature Portfolio Reporting Summary linked to this article.

## Online content

Any methods, additional references, Nature Portfolio reporting summaries, source data, extended data, supplementary information, acknowledgements, peer review information; details of author contributions and competing interests; and statements of data and code availability are available at 10.1038/s41591-023-02419-z.

### Supplementary information

Supplementary Information Supplementary Tables 1–3.

Reporting Summary

## Data Availability

This study is based on publicly available datasets: mortality counts from Eurostat (https://ec.europa.eu/eurostat/statistics-explained/index.php?title=Weekly_death_statistics&stable); temperature values from the European Centre for Medium-Range Weather Forecasts (https://cds.climate.copernicus.eu/cdsapp#!/dataset/reanalysis-era5-land?tab=overview); and population numbers from Eurostat (https://ec.europa.eu/eurostat/cache/metadata/en/demo_r_gind3_esms.htm).

## Code availability

The computer code illustrating the analyses is available at https://github.com/BallesterJoan/europe_summer_2022_heat.
