# Author Correction: Heat-related mortality in Europe during the summer of 2022

**DOI:** 10.1038/s41591-023-02649-1

**Published:** 2023-10-24

**Authors:** Joan Ballester, Marcos Quijal-Zamorano, Raúl Fernando Méndez Turrubiates, Ferran Pegenaute, François R. Herrmann, Jean Marie Robine, Xavier Basagaña, Cathryn Tonne, Josep M. Antó, Hicham Achebak

**Affiliations:** 1 ISGlobal, Barcelona, Spain; 2 Universitat Pompeu Fabra, Barcelona, Spain; 3 Medical School of the University of Geneva, Geneva, Switzerland; 4 Division of Geriatrics, Department of Rehabilitation and Geriatrics, Geneva University Hospitals, Thônex, Switzerland; 5 Molecular Mechanisms in Neurodegenerative Dementia, University of Montpellier, Montpellier, France; 6 École Pratique des Hautes Études, Institut National de la Santé et de la Recherche Médicale, Montpellier, France; 7 PSL Research University, Paris, France; 8 CIBER Epidemiología y Salud Pública, Barcelona, Spain; 9 Institut National de la Santé et de la Recherche Médicale, France Cohortes, Paris, France

**Keywords:** Epidemiology, Health policy

Correction to: *Nature Medicine* 10.1038/s41591-023-02419-z. Published online 10 July 2023.

In the version of this article initially published, there was a typographical error in the second paragraph of the Discussion, where in the text now reading “As a reference, a previous study [*Epidemiology* **34**, e5–e6 (2023)] applied similar epidemiological models to daily temperature and mortality data for Spain only, and found that the summer heat-related mortality burden was 6% higher (that is, 12,054 deaths) than the one reported here…,” 6% originally read “10%” and 12,054 originally read “12,504”. The errors have been corrected in the HTML and PDF versions of the article.

