# Supplementary material for: Heat-related mortality in Europe during the summer of 2022
Source: Nat Med. 2023 Jul 10;29(7):1857–66. doi: 10.1038/s41591-023-02419-z (PMC10353926; doi:10.1038/s41591-023-02419-z)
Supplement: Supplementary file 1 — Supplementary Tables 1–3. [file 41591_2023_2419_MOESM1_ESM.pdf]

nature medicine

Article https://doi.org/10.1038/s41591-023-02419-z

Heat-related mortality in Europe during the summer of 2022

In the format provided by the authors and unedited

Table of contents

Table S1. First-stage sensitivity analysis.
Values correspond to European heat-related mortality numbers during the summer of 2022 (May 30th – September 4th, weeks 22-35) and the whole year of 2022 (all weeks), their corresponding 95% confidence intervals, and the Akaike Information Criterion.

Table S2. European sex- and age-specific heat-related mortality numbers and rates during the summer of 2022.
Summer refers to the 14-week period between May 30th and September 4th of 2022 (weeks 22-35). All-age data by sex was not available in the United Kingdom ([1]). Data by sex and age groups was not available in the United Kingdom, Ireland and Germany ([2]). Values in parenthesis represent the 95% confidence intervals.

Table S3. Second-stage sensitivity analysis.
Significance test for the meta-predictors (p-value), multivariate Cochran Q test for heterogeneity (p-value) and I^2 statistic (%).

Table S1. First-stage sensitivity analysis.

Values correspond to European heat-related mortality numbers during the summer of 2022 (May 30th – September 4th, weeks 22-35) and the whole year of 2022 (all weeks), their corresponding 95% confidence intervals, and the Akaike Information Criterion.

Baseline Model	Attributable Number (deaths) Summer 2022, Weeks 22-35	Attributable Number (deaths) Year 2022, All Weeks	Akaike Information Criterion
Calibration Period: 2015-2019<br>Exposure-Response: Natural Cubic Spline Knots at Percentiles P10, P50, P90	61672 (37643, 86807)	62862 (37935, 88780)	1,623,398

Alternative Models	Attributable Number (deaths) Summer 2022, Weeks 22-35	Attributable Number (deaths) Year 2022, All Weeks	Akaike Information Criterion
Calibration Period: 2015-2022	68476 (44884, 90803)	70997 (46612, 94777)	2,611,328
Exposure-Response: B-Spline of Degree 2	60479 (20606, 100609)	66563 (23778, 109667)	1,626,586
Exposure-Response: Knots at Percentiles P10, P75, P90	58586 (35460, 83392)	59182 (36081, 84254)	1,623,968
Exposure-Response: Knots at Percentiles P10, P25, P75, P90	58802 (31741, 86283)	59496 (32401, 87160)	1,626,818
Exposure-Response: Knots at Percentiles P10, P25, P50, P75, P90	58996 (32829, 84522)	60081 (33672, 85975)	1,630,541

Table S2. European sex- and age-specific heat-related mortality numbers and rates during the summer of 2022.

Summer refers to the 14-week period between May 30th and September 4th of 2022 (weeks 22-35). All-age data by sex was not available in the United Kingdom ([1]). Data by sex and age groups was not available in the United Kingdom, Ireland and Germany ([2]). Values in parenthesis represent the 95% confidence intervals.

	**Attributable Number (deaths)**			**Attributable Rate (deaths per million)**		
**Age Group**	**Women**	**Men**	**Ratio Women / Men**	**Women**	**Men**	**Ratio Women / Men**
0-64 ([2])	2513 (-6226, 9353)	3590 (225, 6540)	0.70	17 (-42, 62)	24 (1, 43)	0.71
65-79 ([2])	4509 (-701, 9030)	4252 (41, 8284)	1.06	144 (-22, 288)	163 (2, 317)	0.88
≥ 80 ([2])	25259 (16442, 34048)	11435 (5766, 16960)	2.21	1819 (1184, 2451)	1432 (722, 2123)	1.27
**All ages ([1])**	**35406 (21576, 46634)**	**21667 (14684, 27998)**	**1.63**	**145 (89, 192)**	**93 (63, 120)**	**1.56**

Table S3. Second-stage sensitivity analysis.

Significance test for the meta-predictors (p-value), multivariate Cochran Q test for heterogeneity (p-value) and I^2 statistic (%).

Model	Meta-Predictor	Wald Test	Q Test	I^2 (%)
Intercept Only	---	---	< 0.001	24.9
Single Predictor	Temperature Average	< 0.001	< 0.001	20.0
	Temperature Interquartile Range	< 0.001	< 0.001	24.5
	Percentage of People Aged 80+	0.029	< 0.001	22.8
Full Model	Temperature Average	< 0.001	< 0.001	17.4
	Temperature Interquartile Range	< 0.001		
	Percentage of People Aged 80+	< 0.001		
